# Visual Navigation Algorithms for Aircraft Fusing Neural Networks in Denial Environments

**DOI:** 10.3390/s24154797

**Published:** 2024-07-24

**Authors:** Yang Gao, Yue Wang, Lingyun Tian, Dongguang Li, Fenming Wang

**Affiliations:** School of Mechatronical Engineering, Beijing Institute of Technology, Beijing 100081, China; 3120225104@bit.edu.cn (Y.G.); 3220225032@bit.edu.cn (L.T.); lidongguang@bit.edu.cn (D.L.); mr_strugglr@163.com (F.W.)

**Keywords:** visual navigation, UAV, denial environment

## Abstract

A lightweight aircraft visual navigation algorithm that fuses neural networks is proposed to address the limited computing power issue during the offline operation of aircraft edge computing platforms in satellite-denied environments with complex working scenarios. This algorithm utilizes object detection algorithms to label dynamic objects within complex scenes and performs dynamic feature point elimination to enhance the feature point extraction quality, thereby improving navigation accuracy. The algorithm was validated using an aircraft edge computing platform, and comparisons were made with existing methods through experiments conducted on the TUM public dataset and physical flight experiments. The experimental results show that the proposed algorithm not only improves the navigation accuracy but also has high robustness compared with the monocular ORB-SLAM2 method under the premise of satisfying the real-time operation of the system.

## 1. Introduction

Unmanned aerial vehicles (UAVs) are widely used in various fields because of their low cost, strong reconnaissance capabilities, flexibility, and maneuverability. Their navigation generally relies on satellite navigation, but in obstructed environments, satellites cannot provide effective support services [1,2]. Therefore, when facing the demands of complex obstructed environments, there is a higher requirement for the navigation technology of future aircraft. Conducting targeted research on visual navigation algorithms for aircraft in obstructed environments is significant.

Conventional visual navigation algorithms are primarily designed for simple indoor environments using established research methods [3,4,5]. However, real-world applications of UAVs often involve intricate outdoor settings with dynamic elements such as pedestrians and vehicles. Consequently, algorithms that excel in static scenarios encounter challenges in maintaining accurate navigation positioning or even experience system failures in the presence of moving objects [6,7]. Additionally, when traditional visual odometry is used on UAV platforms, the computational load and real-time performance are often not fully considered [8,9,10]. Therefore, for UAV on-board edge computing platforms, the main focus should be on ensuring real-time performance while bolstering the resilience of visual odometry in dynamic scenarios.

In recent years, there has been a growing interest in the application of visual odometry for dynamic scenes. The main improvements in this field can be broadly classified into two categories: one based on traditional improvement methods [11,12,13] and the other based on deep learning improvement methods [14,15,16].

Firstly, the traditional improvement-based approach primarily enhances the algorithmic accuracy by analyzing the mapping or clustering relationship between the extracted feature points in order to obtain the static feature points or scenes. Tan et al. [17]. introduced an RD-SLAM method based on a parallel tracking and mapping framework for dynamic scenes. The method identifies the dynamic features of the scene by associating feature points and removes the keyframes of dynamic objects, retaining only the keyframes of static objects. Jaimez et al. [18] proposed the VO-SF algorithm, which uses K-means for image clustering segmentation, performs motion segmentation based on the residuals of pixels on the clustered blocks, and constructs the energy optimization function to regularise the block residuals. Subsequently, Scona et al. [19] enhanced the VO-SF algorithm by incorporating a pyramid optimization framework and generating a static background image through the removal of dynamic objects from the image in real-time, followed by camera pose estimation. The enhanced methodology, which builds upon the traditional approach, can enhance the precision of pose estimation in dynamic settings [20]. However, this approach has the disadvantage of limited real-time performance and requires a static background or initial frame as the main component of the image data. In outdoor UAV flight scenes, the area of moving objects, such as pedestrians and vehicles, may be larger than the background area. Furthermore, there is no guarantee that the initial frame is a static scene, which increases the risk of algorithm failure.

Furthermore, the enhanced deep learning-based approach primarily segments dynamic regions through neural networks, thereby eliminating the impact of dynamic scenes. The development of deep learning techniques has led to the emergence of feasible solutions for target detection and image segmentation. The combination of target detection and image segmentation with visual odometry can be employed for the processing of dynamic scenes utilizing semantic scene information. Berta et al. [21] proposed the DynaSLAM algorithm, which is based on image segmentation using Mask-RCNN for image segmentation and has been integrated with ORB-SLAM2. Jiyu Cheng et al. [22] proposed a dynamic region detection method based on a Bayesian framework to achieve strong robustness. This detection method was proposed as a way to achieve strong robust visual localization. Yong-bao Ai et al. [23] proposed a dynamic visual odometry incorporating YOLOv4 [24], which introduces a target detection method to reduce the influence of dynamic targets on the algorithm. Typically, enhanced deep learning-based methodologies employ image segmentation or two-stage target detection to extract dynamic regions or feature-point-based visual odometry for integration with neural networks, contingent on the necessity for algorithmic precision. This approach also entails a demand for arithmetic capacity on the part of the system’s operational platform. However, for the UAV on-board edge computing platform, the algorithm’s high computational hardware requirements present a challenge to the real-time operation of the system.

This study presents an in-depth analysis of the visual navigation problem in a dynamic UAV environment. A semi-direct visual odometry method [25] with low computational requirements is employed, which strikes a balance between the requirements of navigation accuracy and the real-time operation of the algorithm. The method is combined with a single-stage target detection algorithm to extract and eliminate dynamic objects and reduce their impact on navigation accuracy.

The method proposed in this paper makes the following contributions:

1. A small target detection algorithm is proposed for the viewpoint of unmanned aerial vehicles (UAVs). Given the application background of UAVs and with real-time constraints, we select the YOLOv5 [26], which takes both real-time and accuracy requirements into account, and add the Convolutional Attention Module (CBAM) [27] to enhance the detection accuracy of the algorithm for small targets without increasing the computational cost. Afterward, the correctness of the algorithm selection and the effectiveness of the improvement are verified by comparing it with the YOLOv8 [28] algorithm and YOLOv5 algorithm.
2. The CBAM-YOLOv5 target detection algorithm is combined with semi-direct visual odometry to eliminate dynamic objects in image frames and reduce their impact on navigation accuracy. Furthermore, an improved feature point extraction strategy based on the quadtree algorithm is introduced to enhance its ability and robustness in extracting the background feature points and to reduce the impact of the dynamic region deletion on the feature point extraction.
3. The proposed method was validated in the TUM dataset and actual flight experiments, which demonstrated the effectiveness, usability, and robustness of the proposed algorithm.

## 2. Methods

### 2.1. CBAM-YOLOv5

#### 2.1.1. YOLOv5

**Figure 1:**
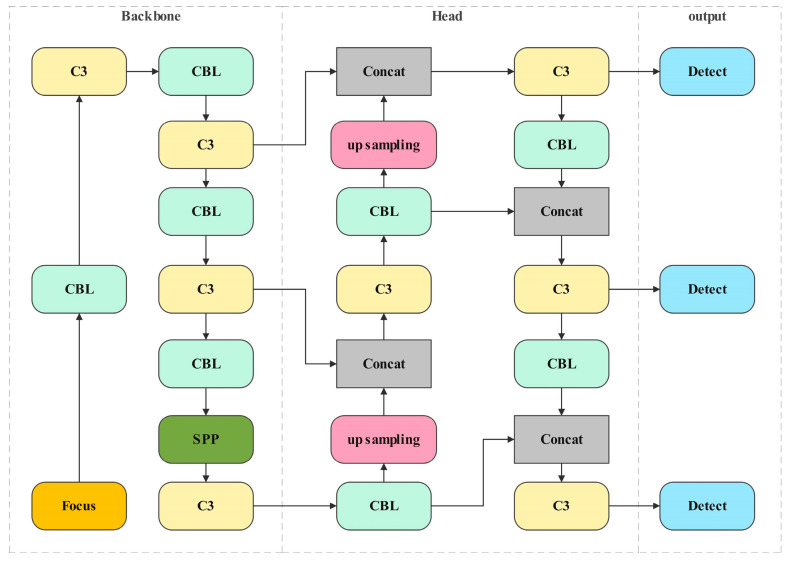
Network of the YOLOv5 [29].

With the remarkable advancements in image processing achieved through deep learning methods, YOLOv5 has emerged as a prominent example of a single-stage object detection algorithm. This approach prioritizes enhanced detection speed over a certain level of accuracy, making real-time detection feasible on edge-computing devices for aerial vehicles. The network structure of YOLOv5 is illustrated in Figure 1.

The network uses RGB images as input and conducts feature extraction through a feature pyramid and a path aggregation network. Subsequently, the extracted features are utilized for prediction through the head, generating bounding boxes, and predicting classes. This study employed pre-training of the YOLOv5s model on the MS COCO dataset for object detection in images captured from a top-down perspective, annotating potential moving objects in the images.

#### 2.1.2. CBAM-YOLOv5 for Small Target Detection on UAV

**Figure 2:**
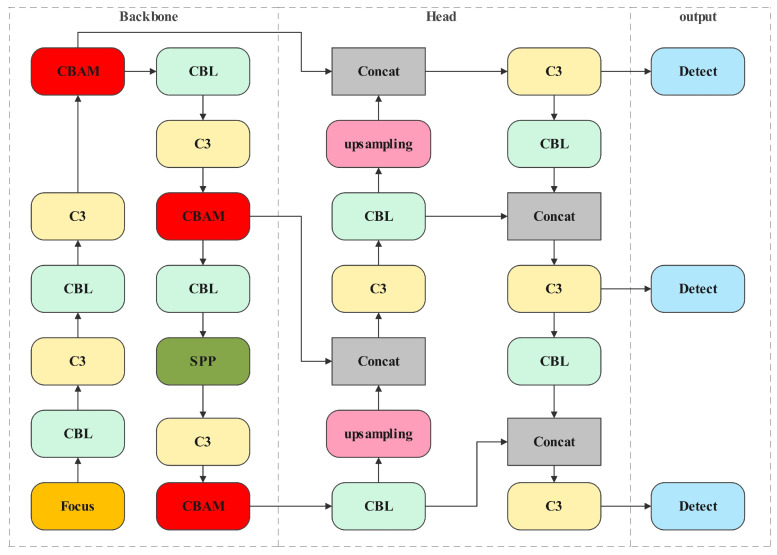
The Network of CBAM-YOLOv5.

In this paper, the focus is on top-down visual odometry images captured from UAVs, with an emphasis on small objects. The YOLOv5 model identified a lack of rich semantic information in the feature maps of the backbone network, as well as insufficient capture of image contours and shallow to mid-level texture information. To address these limitations, the paper proposes integrating the Convolutional Attention Module into the network to enhance crucial information for small object detection. The objective is to improve the network model’s ability to learn spatial and channel features of small and medium-sized objects. The improvement model network is depicted in Figure 2, where the red module is the CBAM module.

#### 2.1.3. Comparison with State-of-the-Art Methods

The CBAM-YOLOv5 algorithm was validated using VisDrone-DET2019 [30]. The training environment consisted of an Ubuntu 18.04 operating system, Intel Core i7-6700k CPU (Intel Corporation, Santa Clara, CA, USA), and NVIDIA GeForce GTX 1070 GPU (NVIDIA, Santa Clara, CA, USA). To facilitate the integration of the algorithm with visual odometry in the future, inference and prediction functions were performed using the NVIDIA Jetson AGX Xavier, which features an 8-core NVIDIA Carmel 64-bit ARMv8.2 CPU and a 512-Core NVIDIA Volta (64 Tensor Cores) GPU. The model evaluation metrics included average precision, recall, and precision; the results are shown in Table 1.

In Table 1, mAP stands for mean average precision and is a metric for evaluating the performance of a target detection algorithm that combines precision and recall to measure the accuracy of a model’s detection of different categories. P stands for precision and R stands for recall. The results indicate that the CBAM-YOLOv5 network model, which incorporates the convolutional attention module, demonstrates improvements in recall, precision, and average precision while maintaining a similar inference speed to the original YOLOv5s model. The experimental results validate the effectiveness of the proposed algorithm. Additionally, although there is a certain gap in accuracy compared to YOLOv8, the limited computational resources of the edge computing platform on the aircraft and the need to ensure real-time performance for subsequent algorithms led to the decision to integrate CBAM-YOLOv5 with the visual odometry algorithm.

### 2.2. Semi-Direct Visual Odometry Fusion with YOLOv5

#### 2.2.1. Algorithm Implementation

##### Basic Principles of Semi-Direct Visual Odometry

The semi-direct method involves estimating the relative camera pose transformation by directly matching local feature blocks of feature points between consecutive frames, as opposed to the direct method, which performs direct matching using the entire image. Although the semi-direct method requires the extraction of image features, its essence is the direct estimation of the pose. In summary, the semi-direct method is a moderate approach that combines the relative advantages and disadvantages of the feature-based and direct methods. In semi-direct visual odometry, the overall task can be divided into two stages: tracking and mapping.

First, the tracking stage estimates the pose of the current frame. Initially, an initial pose estimation was obtained by comparing it with the previous keyframe. Subsequently, by comparing the initial pose with the map, a more accurate pose was obtained, and the observed map points were optimized. Finally, based on keyframe decisions, the seed points of the map are updated by determining whether to update the depth information of the seed points. Second, the mapping stage focuses on estimating the depth information of the feature points [31]. Because monocular visual odometry does not provide depth information, two frames are required to update the depth information of the feature points. When the estimated depth converges, the map points are generated and used by the tracking thread.

##### Semi-Direct Visual Odometry Fusion with YOLOv5

**Figure 3:**
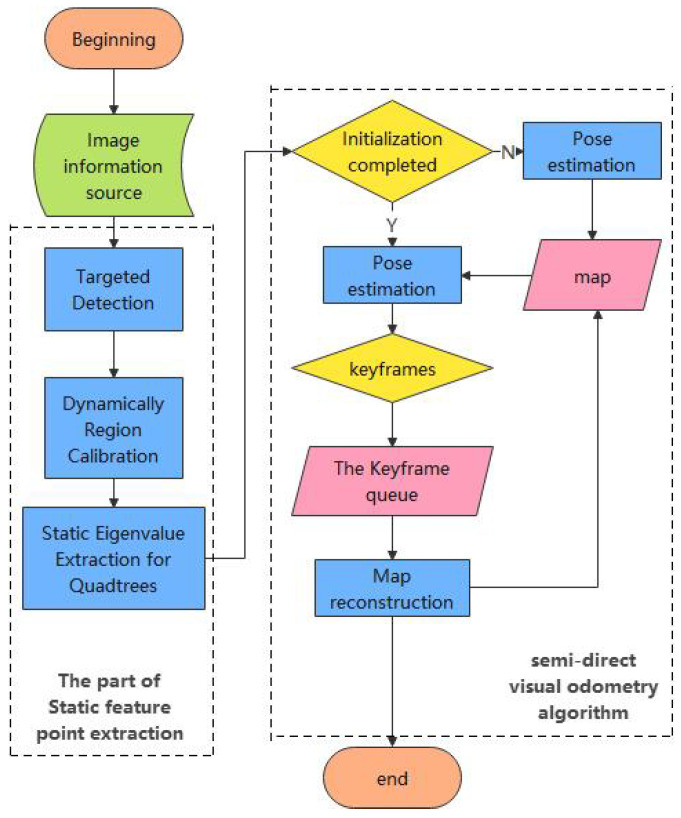
Flow chart of the YOLO-SVO system.

To solve the dynamic scene localization problem in UAV view, the CBAM-YOLOv5 method is combined with the semi-direct visual odometry, thus proposing their fusion method. The method uses CBAM-YOLOv5 to calibrate the dynamic regions of the input image data, performs dynamic region rejection according to the anchor frame position, and performs FAST feature point extraction based on a quadtree structure only in the static regions, so that the feature points on dynamic objects in the scene do not participate in the camera pose solution, eliminating the impact of dynamic scenes on the algorithm accuracy. The method uses the quadtree structure to improve the feature point extraction capability for background and low-texture regions and alleviates the problem of an insufficient number of feature points extracted due to the elimination of dynamic regions. The overall flow of the algorithm is shown in Figure 3.

### 2.3. Static Feature Point Extraction

#### 2.3.1. Dynamic Pixel Calibration

In the context of UAV motion scenarios, challenges arise from significant image disparities caused by rapid device movement or severe shaking during flight, which can exceed the pixel threshold for region matching. To address this issue, semi-direct visual odometry employs an image pyramid approach, in which the original image is progressively scaled down by a constant factor from the bottom-level image to the upper-level image. The tracking results from the previous level serve as the initial estimation for the next level, and the iterative process continues. This method effectively tackles the problem of large image disparities, thereby enhancing the robustness of the algorithm in high-speed motion situations of visual sensors.

**Figure 4:**
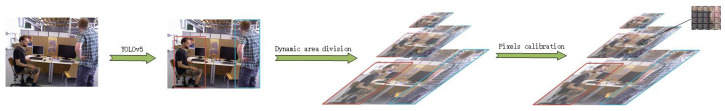
Processing flow of the dynamic pixels marked.

Regarding the image pyramid utilized in semi-direct visual odometry, this study proposes an algorithm that employs the YOLOv5 algorithm to dynamically calibrate the targets in each frame of the original image to obtain the anchor box positions. Using the scaling factor of the image pyramid, the corresponding positions of the anchor boxes at each level were determined, enabling dynamic region partitioning. Dynamic pixel calibration was performed based on the dynamically partitioned regions at each level. Pixels within the dynamic regions at each level of the image pyramid were identified as dynamic pixels, facilitating subsequent feature-point extraction algorithms. Figure 4 shows the processing flow of the dynamic pixels marked.

#### 2.3.2. Static Feature Point Extraction Based on Quadtree

The majority of non-direct visual odometry and SLAM algorithms are feature-based methods such as SVO and monocular ORB-SLAM2. Commonly used feature-point extraction algorithms include SIFT [32], Harris [33], ORB [34], and FAST [35]. The FAST feature point extraction algorithm was employed in semi-direct visual odometry. This feature point extraction algorithm is favored owing to its fast computational speed, making it suitable for real-time algorithms and reducing the inference time. The algorithm proceeds when utilizing the FAST feature point extraction. First, a circular region, denoted as circle(c), was constructed with a fixed pixel radius R centered at point C, and a grayscale difference threshold t was set. Second, an edge point x was selected along the boundary of the circular region, and the grayscale value at this point was compared with the grayscale value at the center pixel, resulting in a grayscale difference. This difference was then compared with the threshold value, t. Finally, these operations were performed for all points within the circular region, and the number of pixels with a grayscale difference greater than the threshold t was recorded. If this count exceeded a predefined value, the center pixel was considered a feature point. After feature point extraction, nonmaximum suppression was applied to the selected feature points. The score of each feature point is calculated based on the sum of the absolute differences between the grayscale values of the center pixel and pixels within the circular region. Only the feature points with maximum response values within the circular region were retained. The FAST feature point algorithm compares grayscale variations between pixels, reducing the computational complexity and improving real-time performance. However, insufficient feature extraction may be encountered in weak-texture regions.

**Figure 5:**
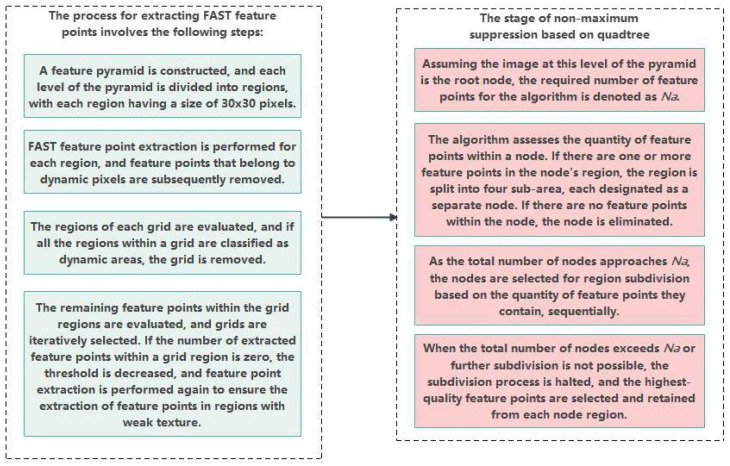
Static feature point extraction.

The static feature point extraction algorithm proposed in this paper encounters challenges when extracting static feature points within a specific region, owing to the incorporation of dynamic pixel calibration in the preconditioning stage. Consequently, the extracted number of feature points fails to meet the minimum threshold required for normal operation of the algorithm. To address this issue, which is based on the quadtree FAST feature point extraction algorithm, ensure that the global number of static feature points meets the algorithm’s operational requirements. This enhancement aims to bolster the robustness of feature-point extraction, particularly in regions with weak textures. The process of static feature-point extraction is illustrated in Figure 5.

**Figure 6:**
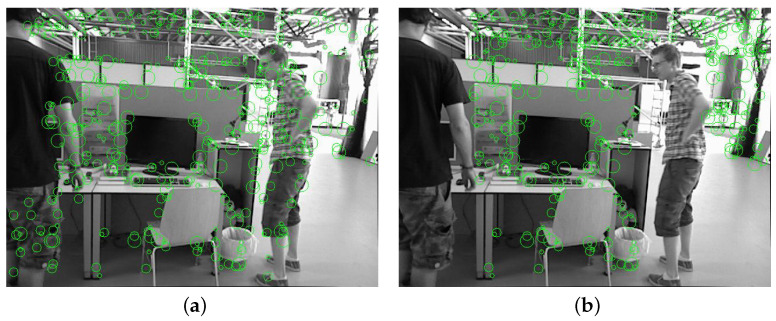
(**a**) The result of the original feature algorithm extraction; (**b**) the result of the static feature algorithm extraction. Comparison of the result among the original feature extraction and the static feature extraction.

The result comparison of two feature point extraction algorithms is illustrated in Figure 6a,b.

They compare the experimental results obtained using the original feature extraction algorithm and the proposed static feature point extraction algorithm presented in this paper. It is evident that the proposed algorithm not only removes feature points on moving objects but also increases the number of feature points extracted from areas with weak textures, such as the background region. This improvement enhances the robustness of the algorithm.

### 2.4. Initial Pose Estimation

UAVs typically capture visual data from a top-down perspective by treating a background as a planar scene. In this scenario, the fundamental matrix (F) and homography matrix (H) play a crucial role. However, when dealing with a planar scene, the fundamental matrix F can encounter degeneracy issues, potentially affecting the accuracy of the algorithm used to solve it. Therefore, the selection of an appropriate matrix is essential to obtain robust and accurate initialization results. This study utilized matrix scoring to evaluate and select matrices. The system calculates the fundamental matrix and homography matrix separately during each run, with the fundamental matrix estimated using the eight-point algorithm, requiring a minimum of eight-point correspondences, and the homography matrix requiring a minimum of four-point correspondences. Once both matrices have been obtained, a matrix evaluation method is employed to determine the type of matrix used for the current pose estimation. The score for the estimated matrix M given a set of point correspondences P can be represented as.

(1) $$S_{M}=\sum_{(p_{i},p_{i}^{\prime })\in P}\left(\rho_{M}\left(d^{2}\left(p,p^{\prime },M\right)\right)\right)$$

In the equation, $\left(p_{i},p_{i}^{\prime }\right)$ represents the successfully tracked feature point correspondences, $d^{2}\left(p,p^{\prime }\right)$ denotes the symmetric transfer error of the two points under the matrix M, and $\rho_{M}\left(d^{2}\right)$ indicates the inlier status of this point correspondence, determined by applying a square root operation.

Finally, the final score of the matrix is determined based on the number of inliers. The determination is calculated according to the following formula.

(2) $$R=\frac{S_{H}}{S_{F}+S_{H}}$$

In the equation, SF represents the score of the fundamental matrix and SH represents the score of the homography matrix. If SH is more significant than 0.4, the homography matrix is selected; otherwise, the basic matrix is selected. Subsequently, the relative camera pose can be obtained by decomposing the matrix chosen, as described in [36].

## 3. Results

### 3.1. The Simulation Experiment

#### 3.1.1. The TUM Dataset

The Technical University of Munich released the TUM dataset [37], which comprises data from 39 sequences captured by a Microsoft Kinect sensor at a rate of 30 Hz. The dataset focuses on including data with dynamic scenes, particularly emphasizing the “freiburg3_walking_” dataset. This dataset contains three sequences with numerous dynamic objects that exhibit varying degrees of motion.

#### 3.1.2. Evaluation of Positioning Accuracy

The two key metrics that are typically considered when evaluating the accuracy of VO are absolute trajectory error (ATE) and relative pose error (RPE). ATE directly measures the disparity between the estimated and ground truth poses, reflecting the accuracy of the algorithm and global trajectory consistency. In contrast, RPE compares the pose error between consecutive frames at fixed time intervals with the ground truth, thus revealing the system’s inherent error. The default time interval for the data in this study was set to 1 s with a unit distance of 1 m.

The experimental platform used was an NVIDIA Jetson AGX Xavier, which can be mounted on a UAV. The power consumption was set to 30 W, and the system environment was Ubuntu 18.04, utilizing GPU acceleration. The experiments involved comparing and analyzing the proposed algorithm with the monocular ORB-SLAM2 [38], DynaSLAM, PL-SVO [39], LEAP-VO [40] and SVO algorithms; the comparative results are presented in Table 2.

**Figure 7:**
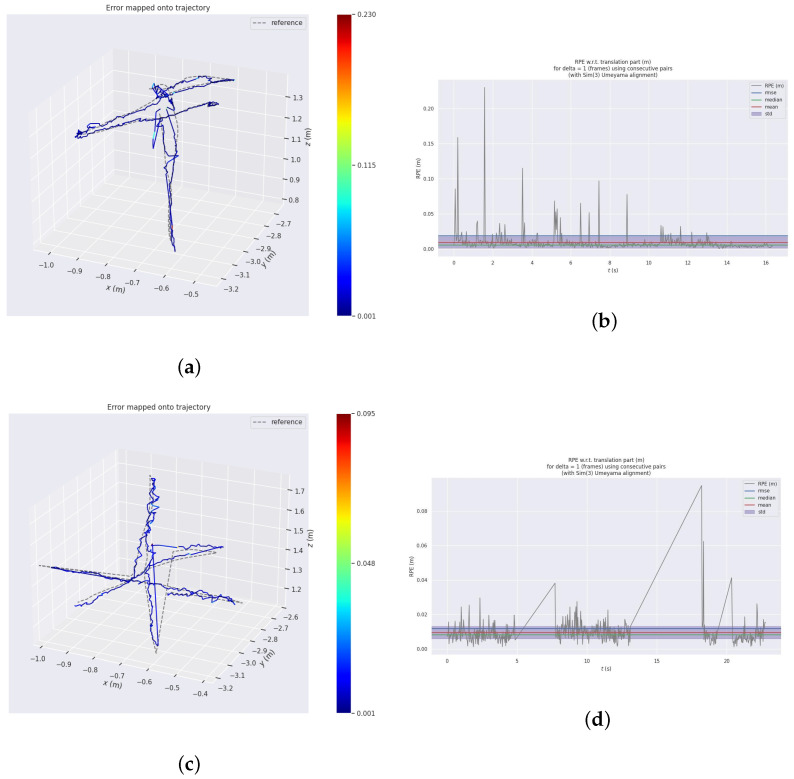
The error of the pose estimation. (**a**) Trajectories parsed by the monocular ORB-SLAM2; (**b**) The RPE of the monocular ORB-SLAM2 parsed trajectories; (**c**) trajectories parsed by our algorithm; (**d**) the RPE of our algorithm’s parsed trajectories.

As shown in Figure 7, we use monocular ORB-SLAM2 for comparative analysis with our algorithm. Figure 7a,c are utilized to illustrate the reconstructed trajectories (colored lines) obtained by applying both the monocular ORB-SLAM2 and the proposed algorithm to the Walking_xyz subset of the TUM dataset fr3 sequence. The reconstructed trajectories were compared with the ground-truth trajectories provided by the dataset (dashed lines), and the relative errors are represented by the colors along the trajectories. Figure 7b,d present the corresponding relative errors of the reconstructed trajectories for the two aforementioned algorithms. As shown in Figure 7a,c, the reconstructed trajectories obtained by the monocular ORB-SLAM2 algorithm are significantly affected by dynamic objects, whereas the algorithm proposed in this paper exhibits smaller relative errors and better alignment with the ground truth trajectories. As depicted in Figure 7b,d, the trajectory reconstruction error of the monocular ORB-SLAM2 algorithm increased significantly in frames with a higher proportion of dynamic objects. In contrast, the proposed algorithm maintains a relatively stable variation in the relative error during trajectory reconstruction owing to the exclusion of the dynamic feature points. The sudden error spikes at 6 s and 15 s are attributed to the relocalization mechanism of the algorithm, which enables rapid and successful relocalization.

Further comparative experiments were conducted using the Walking_* subset of the TUM dataset fr3 sequence to evaluate the performance of the proposed algorithm in dynamic scenarios. The performance of the proposed algorithm is assessed based on the root-mean-square error of the absolute path. The experimental results are listed in Table 2.

The Walking_* subset dataset contains highly dynamic scenes, in which dynamic objects occupy a significant portion of the image. As a result, the monocular ORB-SLAM2 algorithm cannot eliminate all dynamic feature points using bundle adjustment (BA) optimization, leading to significant trajectory reconstruction errors. Compared to the monocular ORB-SLAM2 algorithm, the proposed algorithm achieved an average improvement of 93.55% in trajectory reconstruction accuracy on the Walking_* subset dataset.

Our comparative analysis revealed that algorithms that do not have dynamic object filtering struggle with absolute path estimation, particularly in the presence of dynamic objects and rapid camera motion. However, the performance of the proposed algorithm is outstanding. It effectively excludes dynamic feature points during the feature extraction stage and enhances the quality of feature point extraction through a quadtree structure. This robust approach significantly improves trajectory reconstruction accuracy and instills confidence in its performance.

In most scenes, the proposed algorithms show similar navigation accuracies to DynaSLAM and LEAP-VO algorithms or even exceed them. However, the trajectory reconstruction in the Walking_xyz scene has some limitations compared to DynaSLAM. This is because, compared with DynaSLAM, the proposed algorithm employs object detection algorithms instead of image segmentation algorithms. Although this improves the algorithm runtime and ensures real-time performance, it also increases the calibrated dynamic region and removes more feature points. We will demonstrate the real-time performance later.

The Walking_* subset dataset contains highly dynamic scenes in which dynamic objects occupy a significant portion of the image. Notably, the proposed algorithm does not excel in the trajectory reconstruction. It also demonstrates rapid and successful relocalization, a crucial aspect of its robustness.

#### 3.1.3. Evaluation in Real-Time

Table 3 compares the proposed algorithm’s initialization time and average per-frame processing time with monocular ORB-SLAM2, svo, LEAP-VO and DynaSLAM algorithm. LEAP-VO fails to run after being ported to the edge computing platform. The data in Table 3 show that monocular ORB-SLAM2 has the longest initialization time, while the paper algorithm reduces it by 88.89%. Compared to the semi-direct method, the initialization time increases by 300 ms due to including the YOLOv5 module in the new algorithm.

The paper algorithm’s per-frame processing time, including the object detection algorithm, is 12 ms higher than the semi-direct method. However, it’s important to note that this processing time aligns closely with the inference time of the YOLOv5 algorithm. Furthermore, the paper algorithm demonstrates a per-frame processing time similar to monocular ORB-SLAM2, meeting the crucial real-time requirement of 30 frames per second (FPS) for visual odometry. This validation of its suitability for real-time applications is a significant advantage of the proposed algorithm.

Based on the DynaSLAM algorithm’s time analysis, it can be observed that although it occasionally achieves slightly better trajectory reconstruction accuracy than the proposed algorithm, it fails to meet the real-time requirement for online operation on unmanned aerial vehicle (UAV) platforms. In contrast, the proposed algorithm aligns more closely with practical needs and requirements.

### 3.2. UAV Dynamic Scene Experiment

**Figure 8:**
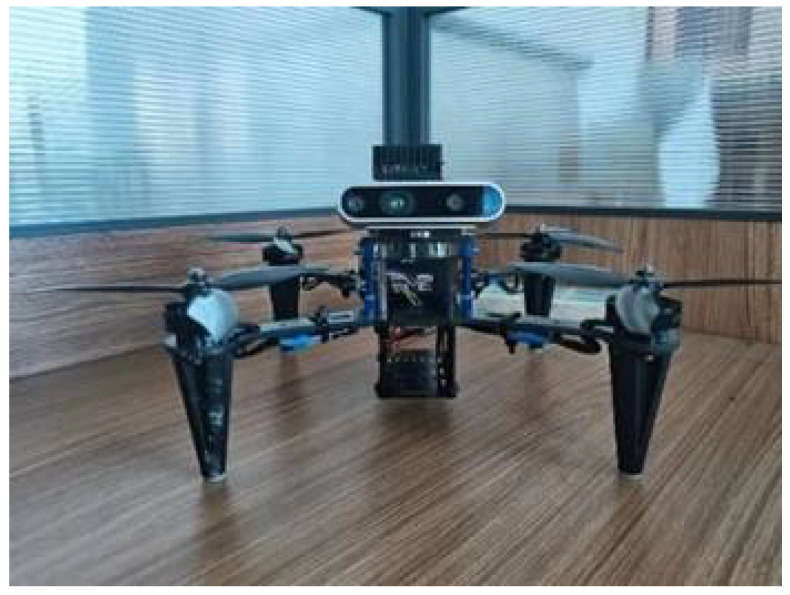
The experimental UAV platform.

**Figure 9:**
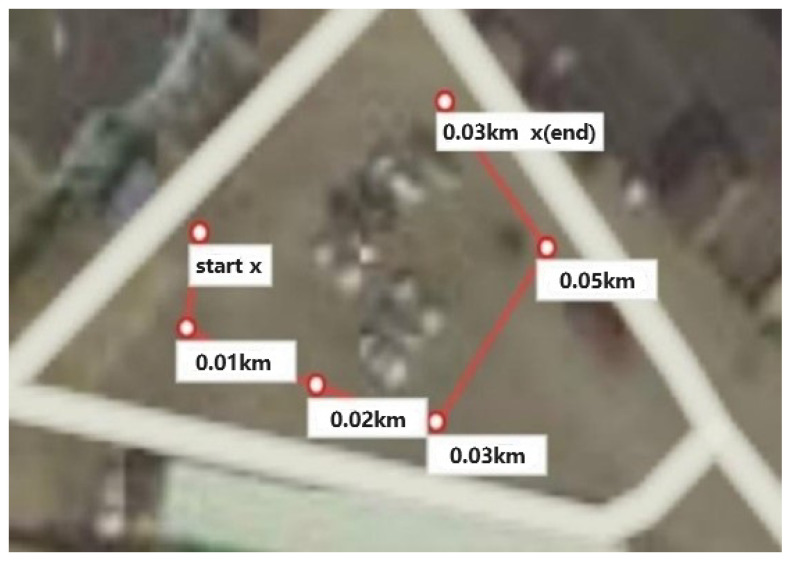
The trajectory of the flight in the real world.

The test platform configured the UAV to assess the visual odometry algorithm’s real-time performance and capabilities. This UAV allowed for visual data collection from the D435i camera (Intel Corporation, USA), inertial measurements from the MPU6000 sensor, and GPS position information. The NVIDIA Jetson AGX Xavier processing unit provided the computational power necessary for real-time data processing and algorithm execution. The Ubuntu 18.04 operating system served as the software platform for seamless integration and control of the UAV system. This experimental UAV platform played a crucial role in conducting dynamic scene experiments, enabling the evaluation and validation of the proposed visual odometry algorithm’s performance and its ability to operate in real-time scenarios on UAV platforms. The testing platform and the flight route are depicted in Figure 8 and Figure 9.

**Figure 10:**
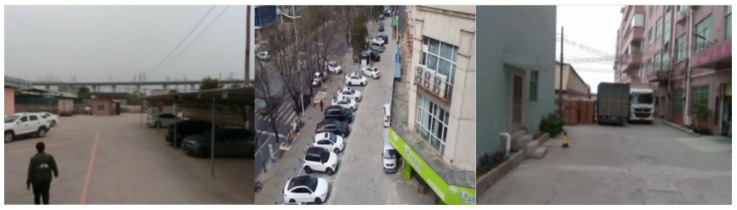
The scene of the flight.

The Xavier processing unit acquired and stored visual data in real-time. During the flight, the proposed algorithm was executed in real-time to analyze the UAV’s flight trajectory while simultaneously storing IMU and GPS data as the standard UAV flight trajectory baseline. After completing the flight experiment, the Xavier processing unit was employed to run monocular ORB-SLAM2 on the captured visual data, obtaining the trajectory analysis results as an experimental reference. The flight experiment took place in an open space beneath residential buildings, as depicted in Figure 10. The standard trajectory data for the experiment were computed using a combination of onboard GPS signals and inertial navigation system data. The visual data encompassed various outdoor scenes, such as cars and pedestrians, which demanded high algorithm robustness. The flight scenes are illustrated in Figure 10.

**Figure 11:**
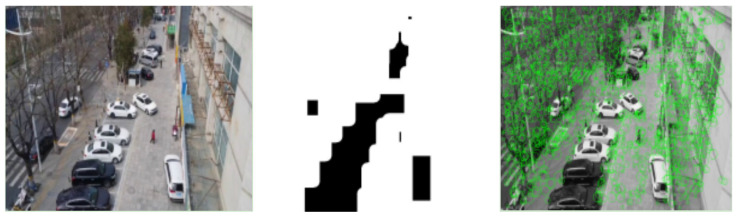
The result of the static feature algorithm extraction.

In Figure 11, from left to right, the RGB original image of the current frame, the mask image after dynamic pixel removal, and the extracted static feature points are shown. It can be observed that the algorithm eliminates feature points located within the quasi-dynamic regions while ensuring a sufficient number of static feature points through the quadtree-based feature point extraction algorithm.

**Figure 12:**
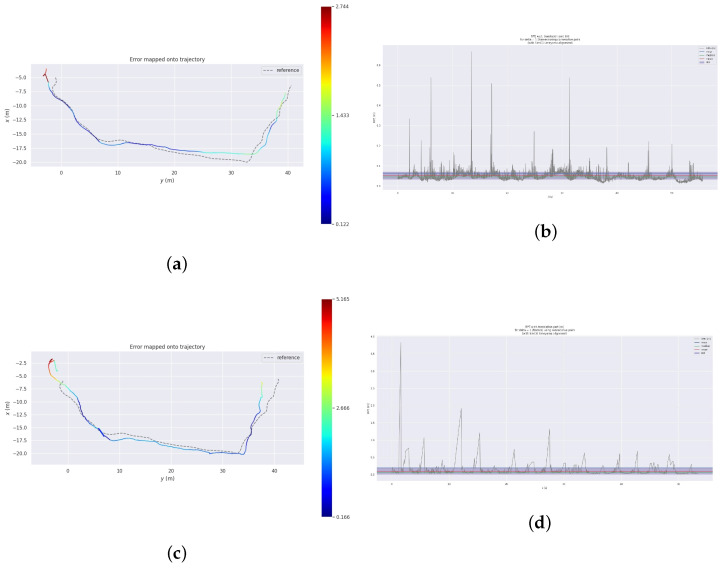
Comparison of the absolute trajectory error. (**a**) Trajectories parsed by our algorithm; (**b**) the RPE of our algorithm-parsed trajectories; (**c**) trajectories parsed by the monocular ORB-SLAM2; (**d**) the RPE of the monocular ORB-SLAM2-parsed trajectories.

Figure 12 is a crucial validation of the proposed algorithm’s effectiveness. Figure 12a,c depicts the trajectory analysis results (colored lines) of the proposed algorithm and the monocular ORB-SLAM2 algorithm for the experimental standard trajectory. The color in the trajectory analysis indicates the magnitude of the absolute error. Figure 12b,d represents the relative errors of the trajectory analysis results of the paper algorithm and monocular ORB-SLAM2 algorithm compared to the actual trajectory. The paper algorithm provides a trajectory analysis closer to the exact trajectory with higher positioning accuracy, thereby validating its superiority.

Dynamic pedestrians and vehicles often lead to discontinuities in trajectory analysis, as observed in the monocular ORB-SLAM2 algorithm. These fluctuations are a significant challenge. However, the proposed improved algorithm tackles this issue head-on by removing dynamic feature points, resulting in a notable improvement in this phenomenon. This adaptability of the paper algorithm is a key strength.

Data analysis reveals that the paper algorithm has a mean square error of 1.03 m for the absolute error of the trajectory analysis and 0.06 m for the relative error of the trajectory. In contrast, the monocular ORB-SLAM2 algorithm has a mean square error of 1.89 m for the absolute error of the trajectory and 0.20 m for the relative error of the trajectory. The paper algorithm achieves a 45.5% improvement in trajectory analysis accuracy compared to the monocular ORB-SLAM2 algorithm. Additionally, the maximum value of the absolute error of the trajectory analysis is reduced by 46.89% in the paper algorithm, demonstrating an enhancement in positioning accuracy.

## 4. Conclusions

This paper addresses the limitations of classical visual odometry algorithms in robustness under dynamic scenes and the inability to ensure real-time performance on onboard edge computing platforms for high-precision algorithms in UAVs. We propose a novel aircraft visual navigation algorithm incorporating neural networks to overcome these challenges. Firstly, we enhance YOLOv5 to improve the accuracy of small object detection, tailored explicitly for UAV visual data features. Experiments demonstrate that the improved algorithm is more suitable for UAV platforms in terms of real-time compared to YOLOv8. Secondly, we fuse the proposed CBAM-YOLOv5 algorithm with a semi-direct visual odometry method to remove dynamic feature points in complex scenes. Thirdly, we employ a combination of quadtree structures to enhance the quality of feature point acquisition. Finally, we validate and evaluate our algorithm using the walking series scenes from the TUM dataset and real-flight scenarios with UAVs. Compared to the monocular ORB-SLAM2 algorithm in dynamic scenes, our proposed algorithm demonstrates significantly improved accuracy, robustness, and real-time performance that meets practical requirements.

In the following steps, we aim to investigate unconventional methods for removing dynamic objects in dynamic scenes and explore algorithms for fast detection and removal of dynamic regions in images. These advancements would further enhance the applicability of our algorithm in real-world outdoor UAV flight scenarios.

## Figures and Tables

**Table 1:** Camparison of the result among the CBAM-YOLOv5 and other typical algorithms.

	mAP	R	P	Inference Time/ms
CBAM-YOLOv5	14.48%	30%	41.43%	7.4
YOLOv5s	13.5%	29.6%	37.8%	7.3
YOLOv8	23%	13.7%	49.8%	27.7

**Table 2:** Comparison of the absolute trajectory error among different algorithms (unit: m).

Fr3 Sequence	ORB-SLAM2	DynaSLAM	PL-SVO	SVO	LEAP-VO	Our
Walking_static	0.102	0.007	0.022	0.009	0.012	0.007
Walking_xyz	0.427	0.015	0.082	0.037	0.030	0.03
walking_rpy	0.741	0.035	0.169	-	0.100	0.04
Walking_halfsphere	0.494	0.025	0.158	0.041	0.029	0.01

**Table 3:** Comparison of initialization time and average processing time of each frame among different algorithms (unit: ms).

fr3 Sequence	ORB-SLAM2		SVO		LEAP-VO		DynaSLAM		Our	
	Initialization	FPS	Initialization	FPS	Initialization	FPS	Initialization	FPS	Initialization	FPS
Walking_static	2732	38.9	120	67.1	-	-	1638	10	325	37.4
Walking_xyz	7415		243						423	
Walking_rpy	4325		223						412	
Walking_halfsphee	6612		132						338	

## Data Availability

Data available on request due to restrictions.
